# A novel rhein-huprine hybrid ameliorates disease-modifying properties in preclinical mice model of Alzheimer’s disease exacerbated with high fat diet

**DOI:** 10.1186/s13578-023-01000-y

**Published:** 2023-03-09

**Authors:** Triana Espinosa-Jiménez, Amanda Cano, Elena Sánchez-López, Jordi Olloquequi, Jaume Folch, Mònica Bulló, Ester Verdaguer, Carme Auladell, Caterina Pont, Diego Muñoz-Torrero, Antoni Parcerisas, Antoni Camins, Miren Ettcheto

**Affiliations:** 1 Department of Pharmacology, Toxicology and Therapeutic Chemistry, Faculty of Pharmacy and Food Science, Universitat de Barcelona, Barcelona, Spain; 2 Institute of Neuroscience, Universitat de Barcelona, Barcelona, Spain; 3 Biomedical Research Networking Centre in Neurodegenerative Diseases (CIBERNED), Madrid, Spain; 4 Department of Pharmacy, Pharmaceutical Technology and Physical Chemistry, Faculty of Pharmacy and Food Sciences, Universitat de Barcelona, Barcelona, Spain; 5 Ace Alzheimer Center Barcelona–International University of Catalunya (UIC), Barcelona, Spain; 6 Institute of Nanoscience and Nanotechnology (IN2UB), Universitat de Barcelona, Barcelona, Spain; 7 Unit of Synthesis and Biomedical Applications of Peptides, IQAC-CSIC, 08034 Barcelona, Spain; 8 Department of Biochemistry and Physiology, Faculty of Pharmacy and Food Sciences, Universitat de Barcelona, Barcelona, Spain; 9 Institute of Biomedical Sciences, Faculty of Health Sciences, Universidad Autónoma de Chile, Talca, Chile; 10 Institut d’Investigació Sanitària Pere Virgili (IISPV), 43201 Reus, Spain; 11 Nutrition and Metabolic Health Research Group, Institute of Health Pere Virgili—IISPV, 43201 Reus, Spain; 12 CIBER Physiology of Obesity and Nutrition (CIBEROBN), Carlos III Health Institute, 28029 Madrid, Spain; 13 Department of Cellular Biology, Physiology and Immunology, Faculty of Biology, Universitat de Barcelona, Barcelona, Spain; 14 Laboratory of Medicinal Chemistry (CSIC Associated Unit), Faculty of Pharmacy and Food Sciences, Universitat de Barcelona, Barcelona, Spain; 15 Institute of Biomedicine (IBUB), Universitat de Barcelona, Barcelona, Spain; 16 Department of Basic Sciences, Universitat Internacional de Catalunya (UIC), Sant Cugat del Vallès, Spain; 17 Unitat de Farmacologia i Farmacognòsia, Facultat de Farmàcia i Ciències de l’Alimentació, Universitat de Barcelona, Av. Joan XXIII 27/31, 08028 Barcelona, Spain

**Keywords:** Rhein-huprine hybrid, Dendritic spines, Cognitive decline, Alzheimer’s disease, High-fat diet, BDNF, BACE1, Neuroinflammation, Tau, TLR4

## Abstract

**Background:**

Alzheimer’s disease (AD) is characterized by a polyetiological origin. Despite the global burden of AD and the advances made in AD drug research and development, the cure of the disease remains elusive, since any developed drug has demonstrated effectiveness to cure AD. Strikingly, an increasing number of studies indicate a linkage between AD and type 2 diabetes mellitus (T2DM), as both diseases share some common pathophysiological features. In fact, β-secretase (BACE1) and acetylcholinesterase (AChE), two enzymes involved in both conditions, have been considered promising targets for both pathologies. In this regard, due to the multifactorial origin of these diseases, current research efforts are focusing on the development of multi-target drugs as a very promising option to derive effective treatments for both conditions.

In the present study, we evaluated the effect of rhein-huprine hybrid (RHE-HUP), a synthesized BACE1 and AChE inhibitor, both considered key factors not only in AD but also in metabolic pathologies. Thus, the aim of this study is to evaluate the effects of this compound in APP/PS1 female mice, a well-established familial AD mouse model, challenged by high-fat diet (HFD) consumption to concomitantly simulate a T2DM-like condition.

**Results:**

Intraperitoneal treatment with RHE-HUP in APP/PS1 mice for 4 weeks reduced the main hallmarks of AD, including Tau hyperphosphorylation, Aβ_42_ peptide levels and plaque formation. Moreover, we found a decreased inflammatory response together with an increase in different synaptic proteins, such as drebrin 1 (DBN1) or synaptophysin, and in neurotrophic factors, especially in BDNF levels, correlated with a recovery in the number of dendritic spines, which resulted in memory improvement. Notably, the improvement observed in this model can be attributed directly to a protein regulation at central level, since no peripheral modification of those alterations induced by HFD consumption was observed.

**Conclusions:**

Our results suggest that RHE-HUP could be a new candidate for the treatment of AD, even for individuals with high risk due to peripheral metabolic disturbances, given its multi-target profile which allows for the improvement of some of the most important hallmarks of the disease.

## Background

Alzheimer’s disease (AD) is defined as a chronic neurodegenerative disease that involves a progressive and irreversible memory loss, followed by a state of total dementia, as well as behavioral disturbances [1, 2]. This neurodegenerative disorder considered the most common form of dementia worldwide [3], displays a high prevalence and increasing incidence, especially among elderly people. In fact, about 33.9 million people worldwide are suffering from AD, and it is expected to triple over the next 40 years [4, 5].

AD is mainly characterized by the presence of abundant extracellular amyloid-beta peptide deposits (Aβ) and intracellular hyperphosphorylated Tau protein (p-Tau), that accumulate to form senile plaques and neurofibrillary tangles (NFTs) respectively, both contributing to neuronal loss [6, 7]. Aβ plaques are produced by the proteolytic cleavages of the amyloid precursor protein (APP) by the beta-secretase 1 (BACE1) enzyme activity and subsequently by γ-secretase, resulting in Aβ peptides of different length, including 38, 40 and 42 amino acids (aa). Specifically, those Aβ composed by 42 aa readily tend to aggregate, resulting in Aβ plaque formation [8, 9]. Phosphorylation is the major modification of Tau protein and it has been described as a critical step in the formation of NFTs [10]. Evidence suggests that Aβ plaques could be involved in the induction of aberrant Tau phosphorylation, thus supporting a causal crosslink between these two pathogenic processes [11–13]. In addition, the aggregation of Aβ into oligomers and fibrils in the brain is also modified by factors such as acetylcholinesterase (AChE), which precipitates the formation of toxic aggregates by accelerating Aβ deposition and increasing its neurotoxicity, contributing to neuroinflammation, oxidative stress and synaptic dysfunction [14, 15]. Additionally, the role of AChE in AD goes much further, since numerous studies have shown the existence of a cholinergic deficit in AD patients due to the modification in the activity of AChE and the decrease in acetylcholine levels [16, 17]. In fact, some of the compounds used as anti-AD drugs like donepezil, galantamine and rivastigmine are AChE inhibitors [18]. However, none of them have been able to totally stop the progression of pathology. For this reason, new approaches to its etiology are being studied nowadays [19]. In addition, it has been described that elevated AChE concentrations could also trigger the systemic inflammation, key in T2DM and AD, representing an interesting therapeutic target for both diseases, which support previous studies that described the possible relationship between AD and metabolic alterations [20–22], stressing AD as a multifactorial disease. In fact, obesity, type 2 diabetes mellitus (T2DM) and metabolic syndrome, all associated with insulin resistance, are recognized risk factors for cognitive disturbances [23–25] and type 3 diabetes has been proposed as a term to describe the complex interlink between insulin resistance and AD [26–28].

Hence, the regulation of metabolic alterations could be an effective strategy to reduce cognitive decline and dementia [29]. In this way, some studies have shown the role of BACE1 in AD progression, not only as a key regulator of the formation of the Aβ peptide but also its function in metabolic regulation [30, 31]. In fact, it has been demonstrated that subtle neuronal expression of human BACE1 resulted in AD phenotypes alongside systemic T2DM-like symptoms, suggesting that BACE1 inhibitors could be used for the treatment of T2DM-associated pathologies [32].

Taken together, evidence suggests that AD is a complex disorder that arises from multiple molecular alterations, therefore, the design of drugs with multiple biological targets could be key for an effective treatment [33]. A recent developed multi-target RHE-HUP hybrid compounds [34] combine the pharmacophores of rhein, a natural product structurally related to some hydroxyanthraquinones with tau anti-aggregating activity, and huprine Y, a strong AChE inhibitor. RHE-HUP displays a strong in vitro activity against its primary targets (tau aggregation and AChE) and a not less strong BACE1 inhibitory activity. Studies conducted in vivo [35] have demonstrated that RHE-HUP reduced Aβ levels, Tau phosphorylation and memory impairment in an APPswe/PS-1dE9 double transgenic mouse model. However, the effect of RHE-HUP on metabolic dysregulation associated to AD has not been evaluated yet. For this reason, the aim of our study was to evaluate the efficacy of this new compound in the progression of AD when it is comorbid with metabolic alterations generated by the chronic consumption of a high-fat diet (HFD).

## Methods

### Animals and treatment

**Fig. 1:**
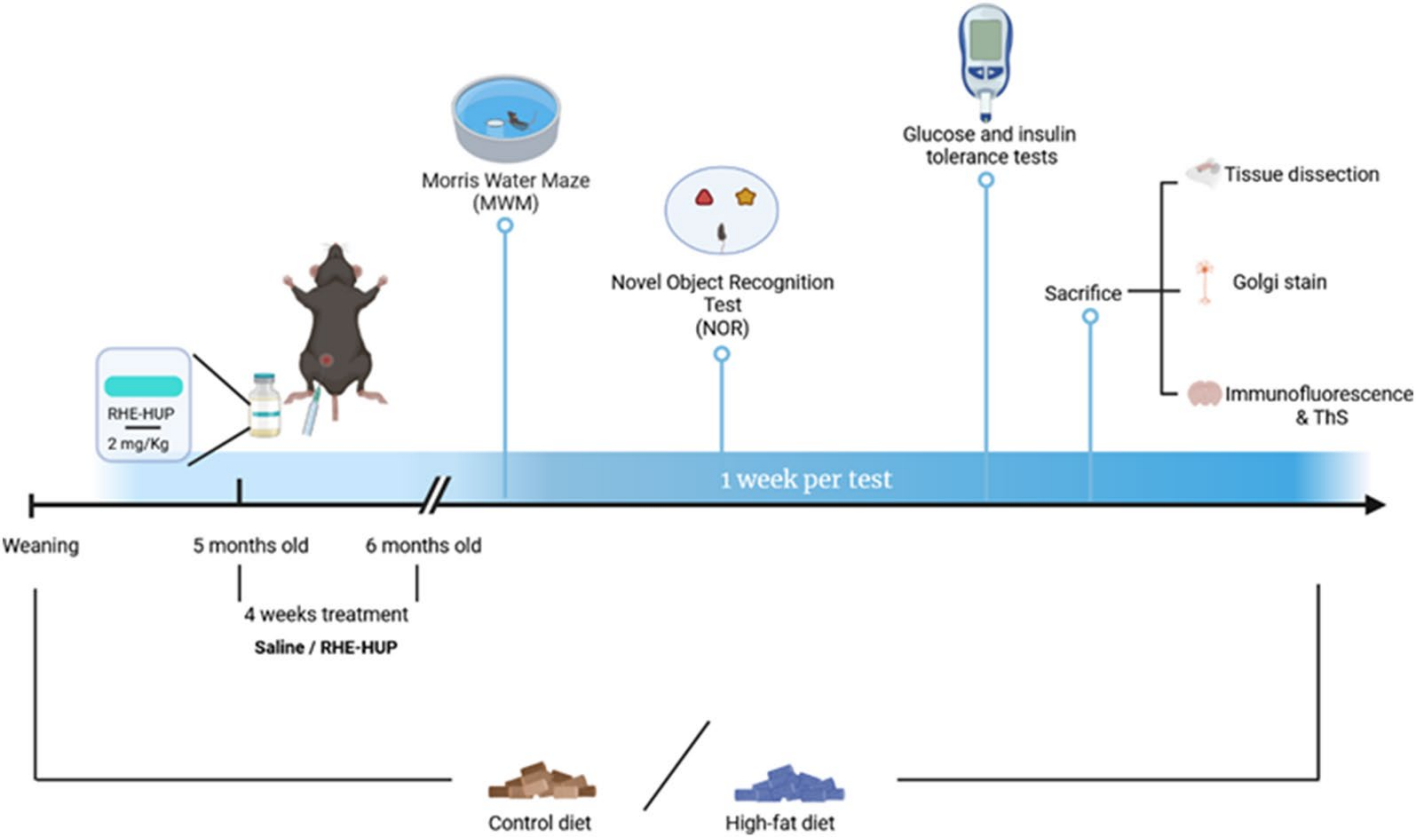
Graphical representation of experimental design. 6 month-old female APP/PS1 and WT littermates were used. After the weaning, animals were fed either control or HFD. When animals were 5 months old, they were treated intraperitoneally (i.p.), either with saline solution or with RHE-HUP at a dose of 2.0 mg/Kg. Then, animals were subjected to two different behavioral tests: MWM and NORT. After that, GTT and ITT were performed and animals were sacrificed by cervical dislocation in order to obtain tissue samples and to perform Golgi Staining Kit, or by intracardially perfusion for immunochemistry/ThS

6 month old female APPswe/PS1dE9 (APP/PS1) double transgenic mice and wild-type (WT) littermates with the same genetic background (C57BL/6) were used. This animal model was chosen according to previous studies reporting that female mice develop higher progressive memory impairment and AD-like neuropathology compared to male mice [36, 37]. These transgenic mice express a Swedish (K594M/N595L) mutation of a chimeric mouse/human APP (mo/huAPP695swe), together with the human exon-9-deleted variant of PS1 (PS1-dE9). In all cases, animals were obtained from established breeding couples in the animal facility (Animal facility from the Faculty of Pharmacy and Food Sciences of the University of Barcelona; approval number C-0032). After the weaning, at 21 days old, and throughout their growth, animals were fed with conventional chow (control diet, CT; ENVIGO, Madison, Wt 53744–4220) or with a palmitic acid-enriched diet containing 60% of fat mainly from hydrogenated coconut oil (HFD) (Research Diets Inc., NB, US). RHE-HUP hybrid (+)-(7R,11R)-N-{9-[(3-chloro-6,7,10,11-tetrahydro-9-methyl-7,11-methanocycloocta[b]quinolin-12-yl)amino]nonyl}-9,10-dihydro-4,5-dihydroxy-9,10-dioxoanthracene-2-carboxamide was prepared as previously reported [38]. When animals were 5 months old, they were treated intraperitoneally (i.p.), either with saline solution or with RHE-HUP at a dose of 2.0 mg/Kg and diluted in bidistilled water with 3% DMSO, three times per week during 4 weeks (Fig. 1). Thus, the study included three experimental groups: WT CT SALINE, APP/PS1 HFD SALINE and APP/PS1 HFD RHE-HUP.

All animals were kept under stable conditions of humidity and temperature, standard light-dark cycle (12 h light/dark cycle) and food and water ad libitum following the ethical guidelines defined by the European Committee (European Communities Council Directive 2010/63/EU). Manipulation protocols were previously approved by the ethics committee from the University of Barcelona, and, at all times, it was made sure that animal numbers, their stress, and pain were kept under a necessary minimum following the appropriate animal manipulation ethical methodologies. All the experiments were performed in accordance with the European Community Council Directive 86/609/EEC and the procedures were established by the Department d’Agricultura, Ramaderia i Pesca of the Generalitat de Catalunya.

### Glucose and insulin tolerance tests

Mice were fasted for 6 h and the tests were performed in a room preheated to + 28 ℃. For the glucose tolerance test (GTT), glucose was administered at a dose of 1 g/Kg i.p. For the insulin tolerance test (ITT), a dose of 0.75 IU/Kg was used. Samples from the tail vein were extracted in consecutive periods. Glucose was measured using an Accu-check^®^ Aviva glucometer at 5, 15, 30, 60 and 120 min after glucose administration and at 15, 30, 45, 60 and 90 min after the insulin administration. To those animals in which blood glucose levels dropped under a concentration of 20 mg/dl in the ITT, a dosage of 1 g/Kg of glucose was administered i.p. 13 animals per group were used.

### Behavioral tests assessments

- Morris water maze (MWM)
  Hippocampal spatial memory and learning memory were assessed by the Morris Water Maze (MWM) test, which was performed as previously reported [39]. Acquired data was analyzed using SMART V3.0 (Panlab Harvard Apparatus, Germany) video tracking system. 13 animals per group were utilized.
- Novel object recognition test (NORT)

NORT was used to assess the hippocampal-dependent recognition memory. 13 animals per group were evaluated in a room with a circular open-field arena of 40 cm in diameter surrounded by black curtains and constant illumination (30 lx) as it has been previously detailed [40]. Data were analyzed by discrimination index (DI) which was calculated using the following equation:

$$ DI\, = \,\frac{B\,exploration\,time\, - \,A\,exploration\,time}{{Total\,exploration\,time}} $$

All spaces were properly cleaned with 96% ethanol between animals, in order to eliminate odor or other cues. Data was measured and represented in seconds.

### Immunoblot analysis

At 6 months, 4–5 animals of each group were sacrificed by cervical dislocation and the liver and hippocampus were dissected and kept at − 80 °C until use. To perform hippocampi and liver extractions, tissues were homogenized in lysis buffer (Tris HCl 1 M pH 7.4, NaCl 5 M, EDTA 0.5 M pH 8, Triton, distilled H20) containing protease and phosphatase inhibitor cocktails (Complete Mini, EDTA-free; Protease Inhibitor cocktail tablets). Total protein concentration was determined using the Pierce^™^ BCA Protein Assay Kit (Thermo ScientificTM). Samples containing 10 µg of protein were analyzed by Western Blot as previously described [41]. Measurements were expressed in arbitrary units and all results were normalized with the corresponding loading control (Glyceraldehyde-3-phosphate dehydrogenase; GAPDH). The used antibodies are detailed in Table 1.

**Table 1:** Primary and secondary antibodies for Western Blotting

Protein	Antibody
ADAM10	ab124695 (abcam)
App	SIG-39152 (Convance)
App C terminal fragment	SIG-39152 (Convance)
DBN1	ABN 207 (Merck Millipore)
GAPDH	MAB374 (Merck Millipore)
GSK3β	#9315 (Cell Signaling Technology)
P-GSK3β (TYR216)	ab75745 (abcam)
IDE	ab32216 (abcam)
IRS2	4502S (Cell Signaling)
Neurexin	ab34245 (abcam)
PTP1B	GTX55767 (Genetex)
sAPPβ	SIG-39138-0 (Covance)
Synaptophisin	M0776 (Dako)
Tau	GTX112981 (Genetex)
P-Tau(ser396)	44752G (Invitrogen)
P-Tau(ser404)	44-758G(Invitrogen)
TLR4	Sc-293072 (Santa Cruz Biotechnology)
Β-actin	A5441 (Sigma)
2nd-ary Goat anti-Rabbit	31460 (Invitrogen)
2nd-ary Goat anti-Mouse	31430 (Invitrogen)

### Enzyme-linked immunosorbent assay (ELISA)

BDNF (Cusabio, China; CSB-E04505m) and amyloid β_1-42_ (ThermoFisher Scientific; kit KHB3441) levels in the cerebral cortex homogenate were detected by ELISA according to manufacturer’s instruction. In both cases, 7 animals per group were analyzed and absorbances were read in a Varioskan LUX Multimode Microplate Reader (Thermo Fisher Scientific). Amyloid β_1-42_ data is expressed in pg/μg protein and BDNF levels are expressed in pg/mg protein.

### β-secretase activity assay kit

Hippocampal tissue from 7 animals were homogenized according to the manufacturer protocol (Abcam; Kit ab282921), and 35 µL of each sample were placed into a 96 well black plate. BACE1 Positive Control and EDANS Standard Curve were also added to the plate. Following the addition of the Reaction Mix, the plate was measured at Ex/Em = 345/500 nm in a kinetic mode for 60 min at 37 °C. Data was treated as specified in the manufacturer’s instructions.

### Immunofluorescence and thioflavin-S staining

15 animals were previously anesthetized by i.p. injection of ketamine (100 mg/Kg) and xylazine (10 mg/Kg). When they were in the no-pain sleep phase, they were intracardially perfused with 4% paraformaldehyde (PFA) diluted in 0.1 M phosphate buffer (PB). After perfusion, brains were removed and stored in 4% PFA at 4 °C overnight (O/N). The next day, the solution was replaced by 4% PFA + 30% sucrose. Coronal sections of 20 μm were obtained by a cryostat (Leica Microsystems, Wetzlar, Germany) and they were kept in a cryoprotectant solution and stored at − 20 °C until use. To perform the experiments, the free-floating technique was used. Briefly, free-floating sections were rinsed in 0.1 M phosphate-buffered saline (PBS) pH 7.35, and after that in PBS-T (PBS 0.1 M, 0.2% Triton X-100). Then they were incubated in a blocking solution (10% fetal bovine serum (FBS), 1% Triton X-100, PBS 0.1 M + 0.2% gelatin) for 1–2 h at room temperature. Later, sections were washed with PBS-T and incubated O/N at 4 °C with the corresponding primary antibody (Table 2). Brain slices were washed with PBS-T and incubated with the corresponding secondary antibody (Table 2) for 2 h at room temperature. Thioflavin-S (ThS) protocol was carried out as previously described [42]. Finally, sections were treated with 0.1 μg/mL Hoechst (Sigma-Aldrich, St Louis, MO, United States), used for cell nuclei staining, for 8 min in the dark at room temperature and washed with 0.1 M PBS. All reagents, containers and materials exposed to Hoechst were properly handled and processed to avoid any cytotoxic contamination. Ultimately, all the samples were mounted in Superfrost^®^ microscope slides using Fluoromount medium (EMS) and were left to dry O/N. Image acquisition was obtained using an epifluorescence microscope (BX61 Laboratory Microscope, Melville, NY OlympusAmerica Inc.) and quantified by ImageJ. 5 animals per group were analyzed.

**Table 2:** Primary and secondary antibodies for Immunofluorescence

Protein	Antibody
GFAP	Z0334 (Dako)
IBA1	O19-19741 (Wako)
2nd-ary Alexa Fluor 488 (Goat-AntiMouse)	A11001 (Life Technologies)
2nd-ary Alexa Fluor 594 (Goat-Anti Rabbit)	A11080 (Life Technologies)

### Hippocampal dendritic spine density analysis

To carry out the spine density analysis, 5 mice in each group were sacrificed by cervical dislocation. Brains were isolated and processed following the instructions of the GolgiStainTM Kit purchased from FD Neurotechnologies, Inc. (FD Rapid GolgiStainTM Kit; Cat #PK401). Images were obtained with a Leica Thunder Microscope (Leica Thunder Imager; Leica Microsystems). The quantification was carried out in 2 different zones, dentate gyrus (DG) and CA1, and 5 neurons per zone and animal were selected. DG was quantified in the secondary branches of the final fragment of the dendrites. In the DG, when analyzing the terminal fragment, 20 µm of dendrite were always left uncounted, and the counting was performed in the following 30 µm. In secondary branches, 20 µm from the ramification were left uncounted and the following 30 µm were analyzed. In CA1, two zones of the neuron were distinguished: CA1 basal and CA1 apical. In CA1 basal, the final part of the dendrite was selected, and again 20 µm of dendrite were always left uncounted, and the counting were performed in the following 30 µm. In CA1 apical, the secondary branches were selected, leaving 20 µm uncounted and analyzing the next 30 µm. Spine density was expressed as the number of spines per 30 μm of dendrite. 5 animals per group were analyzed.

### Statistical analysis

All results are presented as mean ± standard deviation (SD). Normality test was performed, when data followed a parametric distribution and more than two groups were compared, significant differences were determined by one-way analysis of variances (ANOVA), followed by Tukey’s post hoc test for comparison among groups. When only two groups were compared, Student’s t test was performed. However, when data followed a non-parametric distribution, Mann–Whitney and Kruskal–Wallis tests were performed to compare two or more than three groups, respectively. All analyses were obtained using Graph Pad Prism software for Mac version 6.01; Graph Pad Software, Inc.

## Results

### RHE-HUP does not reverse the body weight increase and glucose pathway alterations induced by HFD at peripheral level

**Fig. 2:**
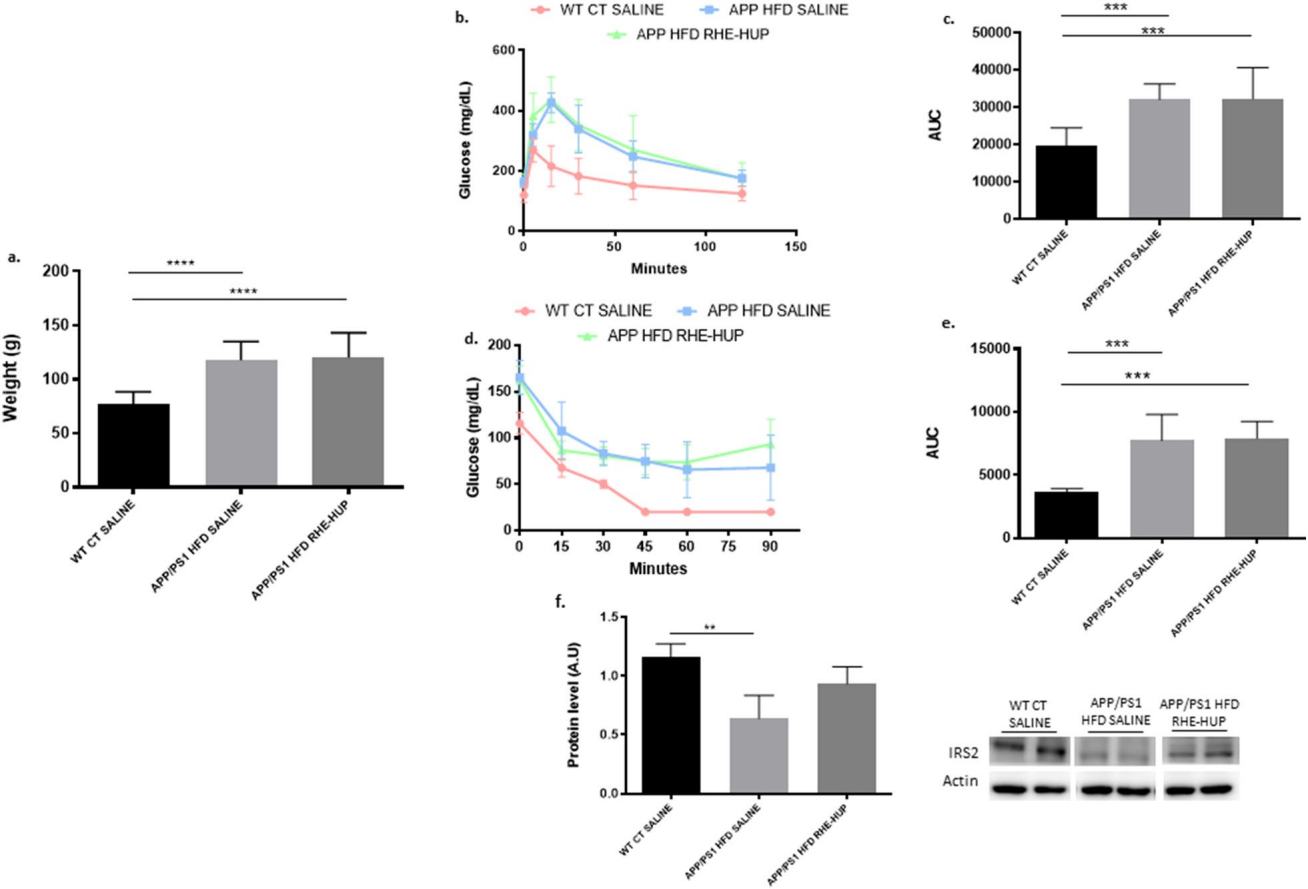
**a**. Analysis and representation of changes in body weight (n = 13 animals per group). **b**. GTT and **d**. ITT experiment profiles (n = 13 animals per group). Area under curve (AUC) data were calculated from the time point 0 until the end of the experiment for both **c**. GTT and **e**. ITT. **f**. Semi-quantification of IRS2 levels in the liver where two representative samples out of four or five per group are shown (n = 4–5). All results were represented as mean ± SD. Statistical analysis was conducted through one-way ANOVA and Tukey post-test, except in the case of the analysis of weights, where the Kruskal–Wallis test was performed. In all cases, ** p < 0.01, *** p < 0.001 and **** p < 0.0001

As it has been widely described, the consumption of HFD is related to the increase in body weight, as well as to hyperglycemia and insulin resistance in mice [43, 44]. As expected, animals following a HFD showed a significant 6 month increased body weight compared with WT CT SALINE group (p < 0.0001) (Fig. 2a). The RHE-HUP treatment did not attenuate the weight gain induced by the HFD. Regarding glucose and insulin metabolism, HFD feeding showed a significant effect in both GTT (WT CT SALINE vs APP/PS1 HFD SALINE p < 0.001; WT CT SALINE vs APP/PS1 HFD RHE-HUP p < 0.001) and ITT (WT CT SALINE vs APP/PS1 HFD SALINE p < 0.001; WT CT SALINE vs APP/PS1 HFD RHE-HUP p < 0.001), regardless of the treatment (Fig. 2b–e). Because the insulin receptor substrate protein 2 (IRS2) is a key target in the hormonal control of metabolism, we measured the hepatic IRS2 protein level. A significant decrease in APP/PS1 HFD SALINE compared with WT CT SALINE (p < 0.01) was detected. However, no significant reduction was observed after the RHE-HUP treatment (Fig. 2f) suggesting that RHE-HUP does not regulate metabolic alterations observed after HFD consumption.

### RHE-HUP treatment improves brain insulin signaling and attenuates Tau hyperphosphorylation

**Fig. 3:**
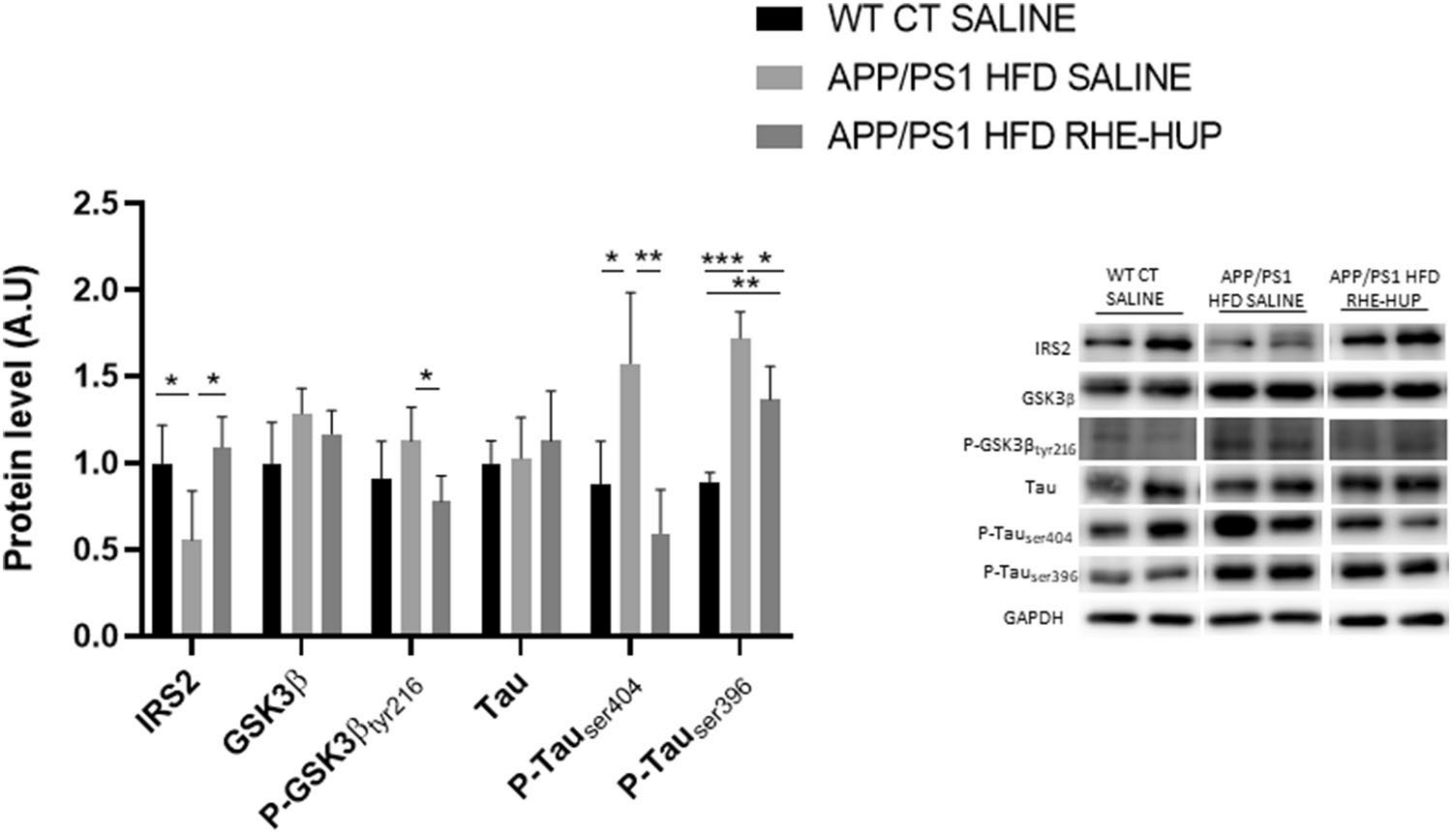
Semi-quantification of hippocampal insulin signaling pathway related proteins and Tau. Two representative samples out of four or five per group are shown (n = 4–5). All results were represented as mean ± SD. Groups were compared against each other using one-way ANOVA and Tukey post-test, except in the case of Tau protein, where Kruskal–Wallis was performed. In all cases, * p < 0.05, ** p < 0.01 and *** p p < 0.01

Alterations in the insulin signaling pathway have been observed in brains of AD patients [45, 46], in which IRS2 represents an important component. Our results demonstrated that the hippocampal levels of IRS2 were significantly decreased in the group APP/PS1 HFD SALINE compared with the control group (p < 0.05). Surprisingly, a recovery in IRS2 was observed after RHE-HUP treatment (p < 0.05) (Fig. 3). Since the increase in IRS2 levels has been related with an attenuation in Tau hyperphosphorylation [47], we evaluated the glycogen synthase kinase-3β (GSK3β), a main Tau kinase converging between AD and insulin resistance. Our results displayed a non-significant upward trend in the group APP/PS1 HFD SALINE when compared with WT CT SALINE. By contrast, those animals treated with RHE-HUP showed a significant decrease of GSK3β phosphorylation levels in tyrosine 216 when compared to the APP/PS1 HFD SALINE mice (p < 0.05) (Fig. 3). Regarding Tau phosphorylation in the hippocampus, our results showed a significant increase in P-Tau levels at serine 404 and serine 396 in APP/PS1 HFD SALINE mice when comparing with WT CT SALINE (P-Tau_ser404_ p < 0.05; P-Tau_ser396_ p < 0.001) and this effect was significantly reduced after RHE-HUP treatment (P-Tau_ser404_ p < 0.01; P-Tau_ser396_ p < 0.05). Our data did not show any significant changes in total Tau protein levels (Fig. 3).

### RHE-HUP reduces Aβ plaques by regulating APP processing and Aβ degradation in APP/PS1 mice fed with HFD

**Fig. 4:**
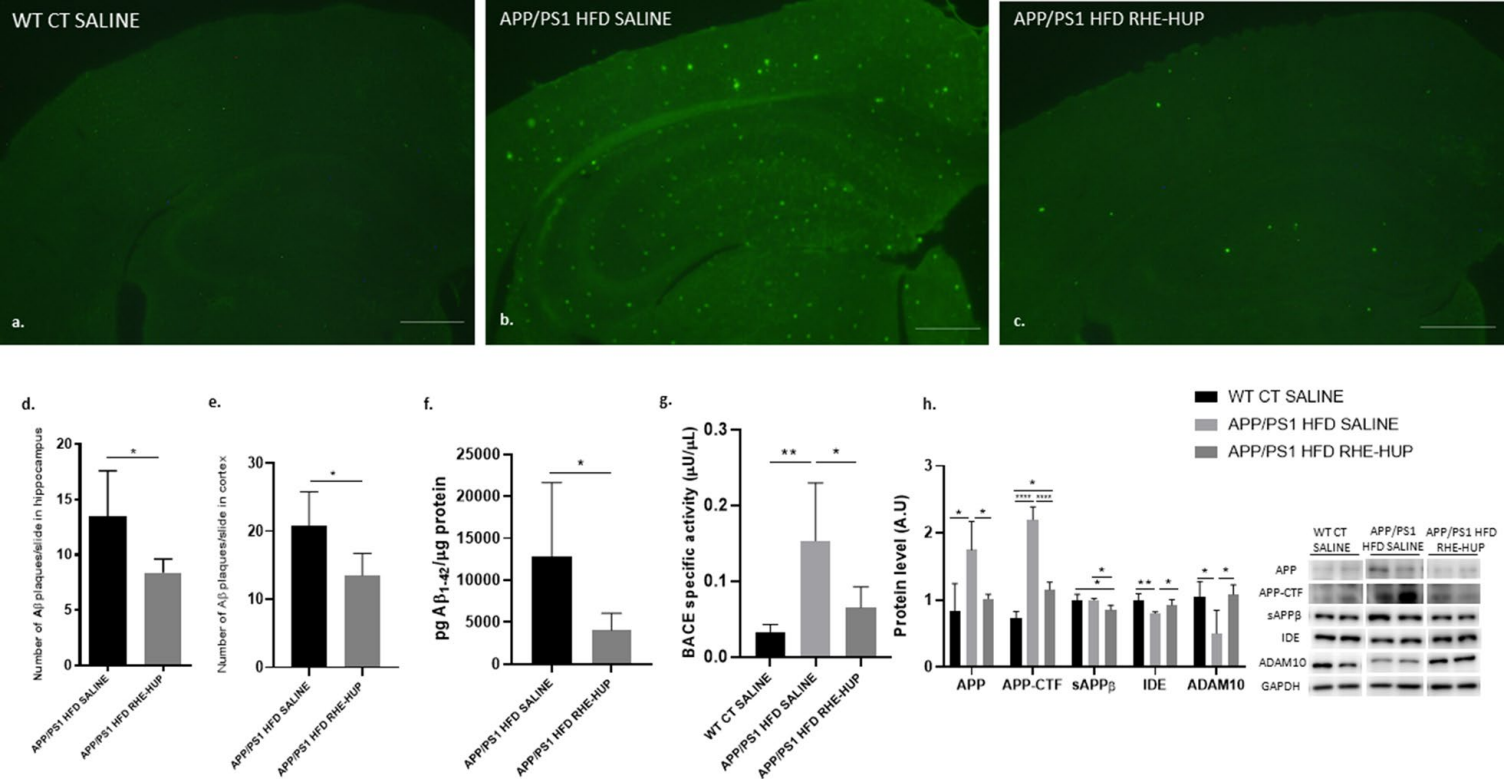
**a**–**c**. Illustrative images of Aβ plaques in the hippocampus and cortex. Scale bar: 200 µm. Graphic representation of Aβ plaques quantification in **d**. hippocampus and **e**. cortex (n = 5 independent samples per group, with at least 5 slices analyzed per sample). In hippocampus analysis, Mann–Whitney test was performed, * p < 0.05. In cortex analysis, t-test was performed, where * p < 0.05. **f**. Measurement of the levels of Aβ_42_ peptide in the cortex (n = 7). Statistical analysis was performed by T-test, where * p < 0.05. **g**. Determination of β-secretase activity in the hippocampus (n = 7). Data were analyzed by one-way ANOVA and Tukey’s post-test, where * p < 0.05 and ** p < 0.01 **h**. APP processing related protein levels. Two representative samples out of four or five per group are shown (n = 4–5). All results were represented as mean ± SD. Groups were compared against each other using one-way ANOVA and Tukey post-test, * p < 0.05, ** p < 0.01 and **** p < 0.0001

To assess the state of Aβ burden in the hippocampus and cortex, ThS was used for detection of senile plaques. Our results demonstrated a significant decrease in the number of plaques after treatment in both regions, as shown in the images (Fig. 4a–c) and in the graphic representation (p < 0.05) (Fig. 4d–e). This result was corroborated with the significant reduction of Aβ (1–42) levels (p < 0.05) observed in the cortex after RHE-HUP administration (Fig. 4f). To elucidate the mechanisms by which RHE-HUP induced Aβ reduction, the analysis of APP processing and Aβ degradation was performed. Regarding the first one, full-length APP was analyzed. As expected, non-treated transgenic mice showed a significant increase in this protein level (p < 0.05) whereas these levels were reduced in those animals treated with RHE-HUP (p < 0.05) (Fig. 4h). In this line, BACE1 activity also showed a significant increase in APP/PS1 HFD SALINE when compared with WT CT SALINE (p < 0.01) and decreased after treatment (p < 0.05) (Fig. 4g).

APP-C-terminal fragment (APP-CTF) was significantly increased in non-treated transgenic mice compared to control group whereas soluble amyloid precursor protein β fragment (sAPPβ) did not show differences in WT vs APP/PS1 HFD. However, both proteins were reduced after treatment (APP-CTF: WT CT SALINE vs APP/PS1 HFD RHE-HUP p < 0.05; APP/PS1 HFD SALINE vs APP/PS1 HFD RHE-HUP p < 0.0001; sAPPβ: WT CT SALINE vs APP/PS1 HFD RHE-HUP p < 0.05; APP/PS1 HFD SALINE vs APP/PS1 HFD RHE-HUP p < 0.05).

The insulin-degrading enzyme (IDE) is one of the main proteases involved not only in the degradation of insulin but also in that of Aβ peptide [48]. Our results showed a significant reduction in the hippocampus of APP/PS1 HFD SALINE mice compared to WT CT SALINE, levels which were recovered after RHE-HUP treatment (WT CT SALINE vs APP/PS1 HFD SALINE p < 0.01; APP/PS1 HFD SALINE vs APP/PS1 HFD RHE-HUP p < 0.05).Similarly, ADAM10, a neuroprotective protein involved in the non-amyloidogenic pathway, experimented a significant reduction in APP/PS1 HFD SALINE (p < 0.05) when compared with WT CT SALINE mice, levels that were recovered after RHE-HUP treatment, reaching values similar to those of controls (p < 0.05) (Fig. 4h).

### RHE-HUP treatment decreases glial reactivity in APP/PS1 HFD mice

**Fig. 5:**
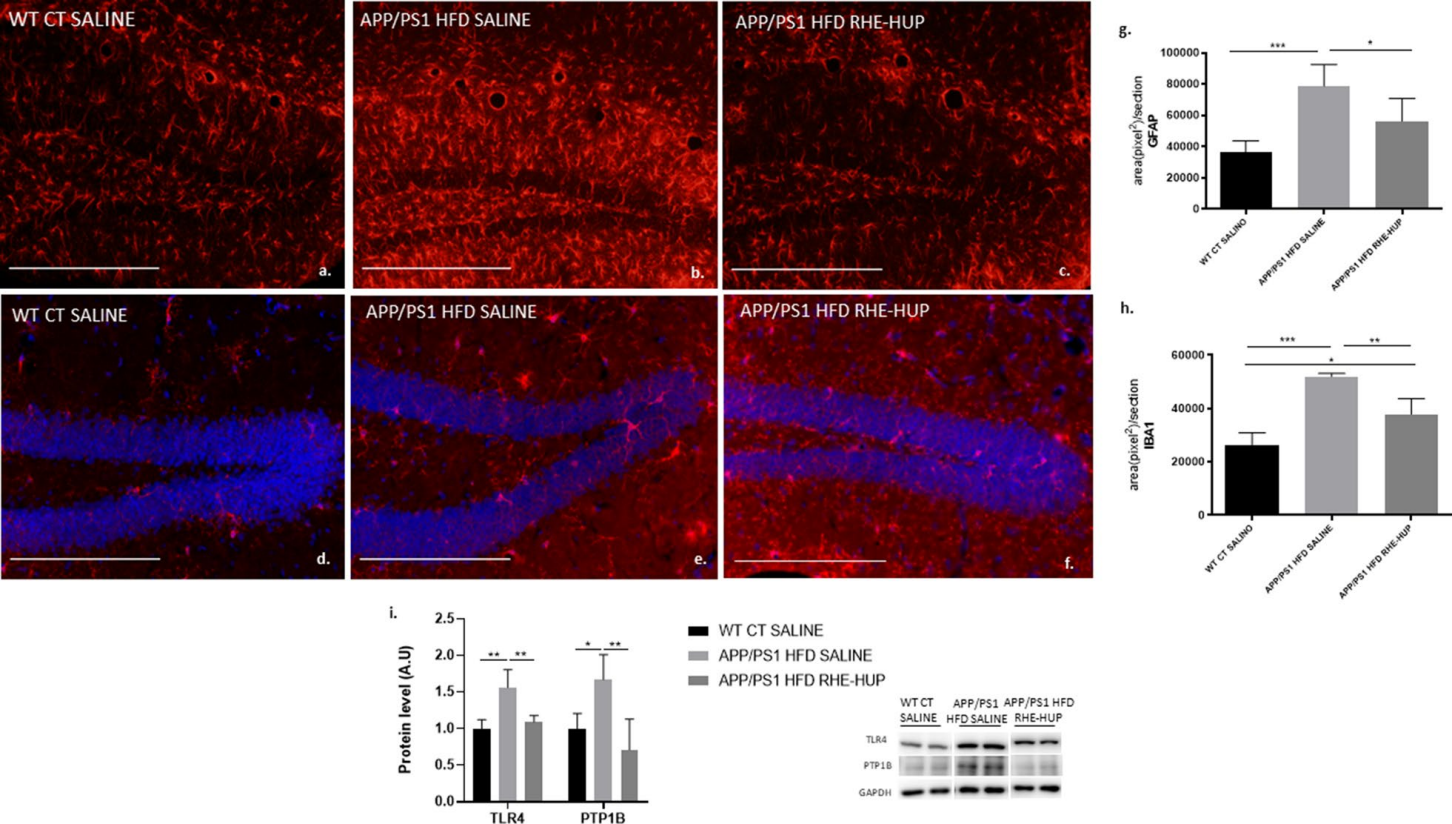
Evaluation of inflammatory responses. Representative images for the detection of astrocytes **a**–**c**, and microglia **d**–**f**, co-stained with Hoechst for the detection of cellular nucleus (blue). Scale bar: 200 µm. Graphic representation of fluorescence intensity quantification for GFAP **g** and IBA1 **h**. In both cases, statistical analysis was performed through one-way ANOVA (n = 5) and Tukey’s post hoc test, * p < 0.05. ** p < 0.01 and *** p < 0.001. **i.** protein levels for TLR4 and PTP1B where two representative samples out of four or five per group are shown (n = 4–5). All results were represented as mean ± SD. Groups were compared against each other using one-way ANOVA and Tukey post-test, * p < 0.05 and ** p < 0.01

Increasing evidence correlates neuroinflammation with the development of AD [49, 50]. In our study, the evaluation of astrocytes and microglial reactive profile was studied in the dentate gyrus of the hippocampus by detecting glial fibrillary acidic protein (GFAP) and ionized calcium-binding adapter molecule 1 (IBA1), astrocyte and microglial markers, respectively (Fig. 5a–f). Our results showed a glial activation in those transgenic animals fed with HFD compared to WT and a clear reduction of this reactivity after the RHE-HUP treatment. These results were corroborated by the fluorescence intensity quantification data. A significant increase in astrogliosis and microglial activation in transgenic mice fed with HFD in comparison to the WT CT SALINE groups was found (p < 0.001). By contrast, this increase was significantly attenuated when these animals were treated with RHE-HUP (GFAP: APP/PS1 HFD SALINE vs APP/PS1 HFD RHE-HUP p < 0.05; IBA1: APP/PS1 HFD SALINE vs APP/PS1 HFD RHE-HUP p < 0.01) (Fig. 5 g–h).

Toll-like receptor 4 (TLR4) and protein tyrosine phosphatase (PTP1B), both related with neuroinflammation, were analyzed in the hippocampus. In agreement with glial profile, our results showed a similar pattern where concentrations of both proteins were significantly increased in the APP/PS1 HFD SALINE group compared to WT CT SALINE (TLR4: p < 0.01; PTP1B: p < 0.05), returning to baseline levels after treatment with RHE-HUP (p < 0.01, in both cases) (Fig. 5i).

### RHE-HUP increases dendritic spines density and synaptic biomarkers in APP/PS1 HFD mice

**Fig. 6:**
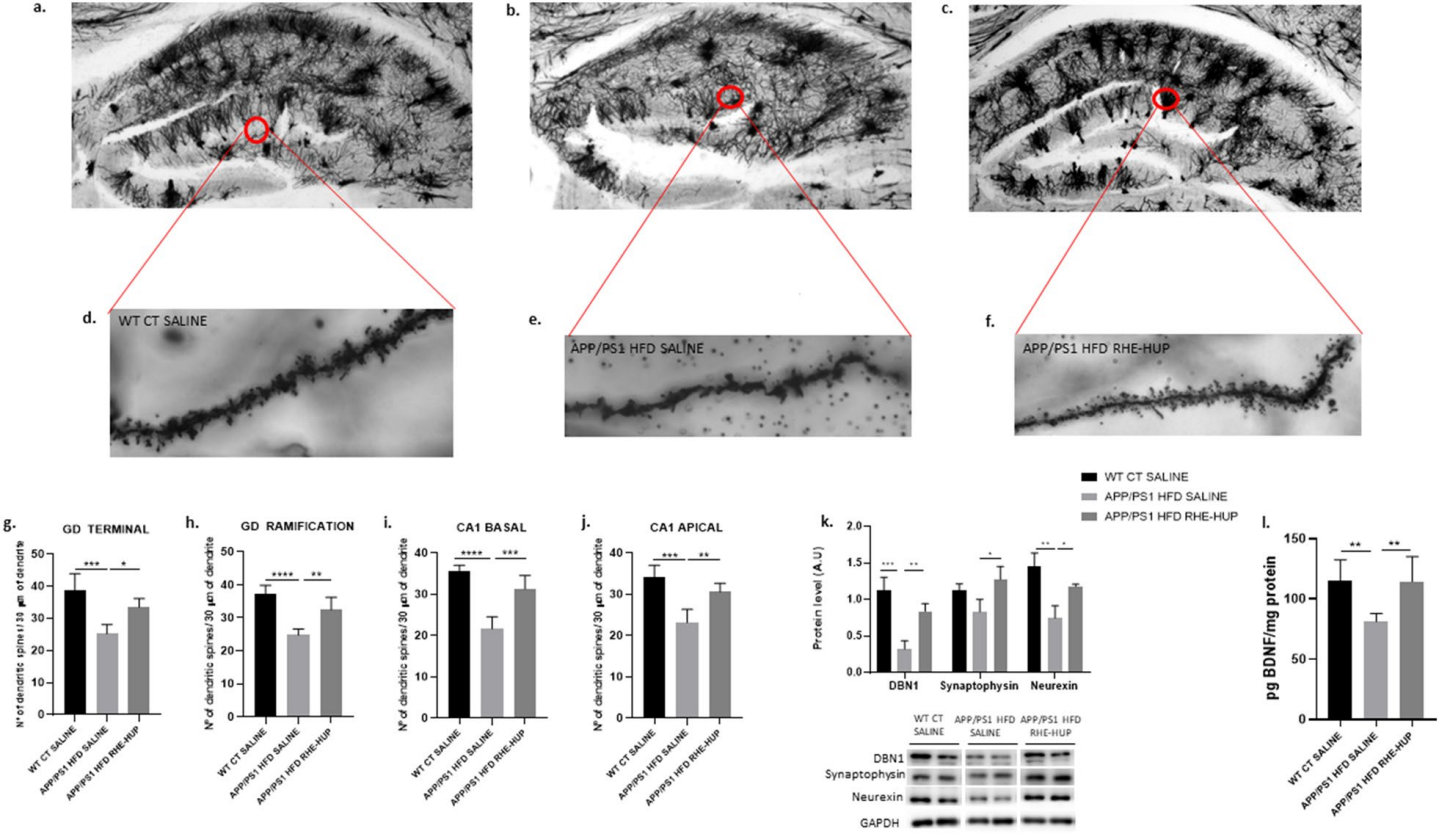
Optical microscope images of the hippocampus **a**–**c** and representative magnification images of dendritic spines of each experimental group **d**–**e**. **g**–**j**. Quantification of dendritic spines of each 30 µm of dendrite in different areas of the hippocampus (n = 5). Groups were compared against each other using one-way ANOVA and Tukey post-test, * p < 0.05, ** p < 0.01, *** p < 0.001 and **** p < 0.0001 k. Representative images of synaptic proteins levels were determined, where two representative samples out of four or five per group are shown (n = 4–5). Graphs barts represent mean ± SD. Data were analyzed by one-way ANOVA and Tukey’s post-test, * p < 0.05, ** p < 0.01 and *** p < 0.001. **l**. Quantification of BDNF protein levels in the cortex (n = 7). Data were analyzed by one-way ANOVA and Tukey’s post-test, ** p < 0.01

The reduction in the number of dendritic spines together with alterations in cognition has been widely demonstrated in AD patients, suggesting that they could play a key pathogenic role [51, 52]. Optical microscope images of the hippocampus are shown in Fig. 6a–c, accompanied by a representative magnification image of dendritic spines of each experimental group (Fig. 6d–f). A significant decrease in the number of dendritic spines was observed in APP/PS1 HFD SALINE when comparing with the control group (Fig. 6g–j), while in those animals treated with RHE-HUP, this reduction was reverted reaching levels similar to the control regardless of the studied area in the hippocampus (**DG TERMINAL:** WT CT SALINE vs APP/PS1 HFD SALINE p < 0.001; APP/PS1 HFD SALINE vs APP/PS1 HFD RHE-HUP p < 0.05. **DG RAMIFICATION:** WT CT SALINE vs APP/PS1 HFD SALINE p < 0.0001; APP/PS1 HFD SALINE vs APP/PS1 HFD RHE-HUP p < 0.01. **CA1 BASAL:** WT CT SALINE vs APP/PS1 HFD SALINE p < 0.0001; APP/PS1 HFD SALINE vs APP/PS1 HFD RHE-HUP p < 0.001. **CA1 APICAL:** WT CT SALINE vs APP/PS1 HFD SALINE p < 0.001; APP/PS1 HFD SALINE vs APP/PS1 HFD RHE-HUP p < 0.01.).

Different synaptic proteins involved in memory process and plasticity, such as drebrin 1 (DBN1), synaptophysin and neurexin, were measured by Western Blot. Our results showed a significant decrease in DBN1 protein levels in the APP HFD SALINE group when they were compared with the control group (p < 0.001), while DBN1 levels were rescued after RHE-HUP administration (p < 0.01). A similar pattern was observed for the other synaptic proteins studied, but in the case of synaptophysin the values did not reach statistical significance, and only a positive trend was observed (Synaptophysin: APP/PS1 HFD SALINE vs APP/PS1 HFD RHE-HUP P < 0.05; Neurexin: WT CT SALINE vs APP/PS1 HFD SALINE p < 0.01; APP/PS1 HFD SALINE vs APP/PS1 HFD RHE-HUP p < 0.05) (Fig. 6k).

Moreover, one protein that deserves special mention is BDNF plays a critical role not only in the growth and development of the nervous system, but also as a modulator of synaptic plasticity, suggesting that its regulation could play a key role in the preservation of cognitive function [53]. In this line and, in accordance with the results shown above, the analysis of BDNF levels in the cortex demonstrated a significant decrease in APP/PS1 HFD SALINE in comparison with WT CT SALINE (p < 0.01). Nevertheless, the treatment with RHE-HUP resulted in an increase of BDNF (p < 0.01) (Fig. 6l).

### The treatment with RHE-HUP improves the cognitive process in APP/PS1 HFD mice

**Fig. 7:**
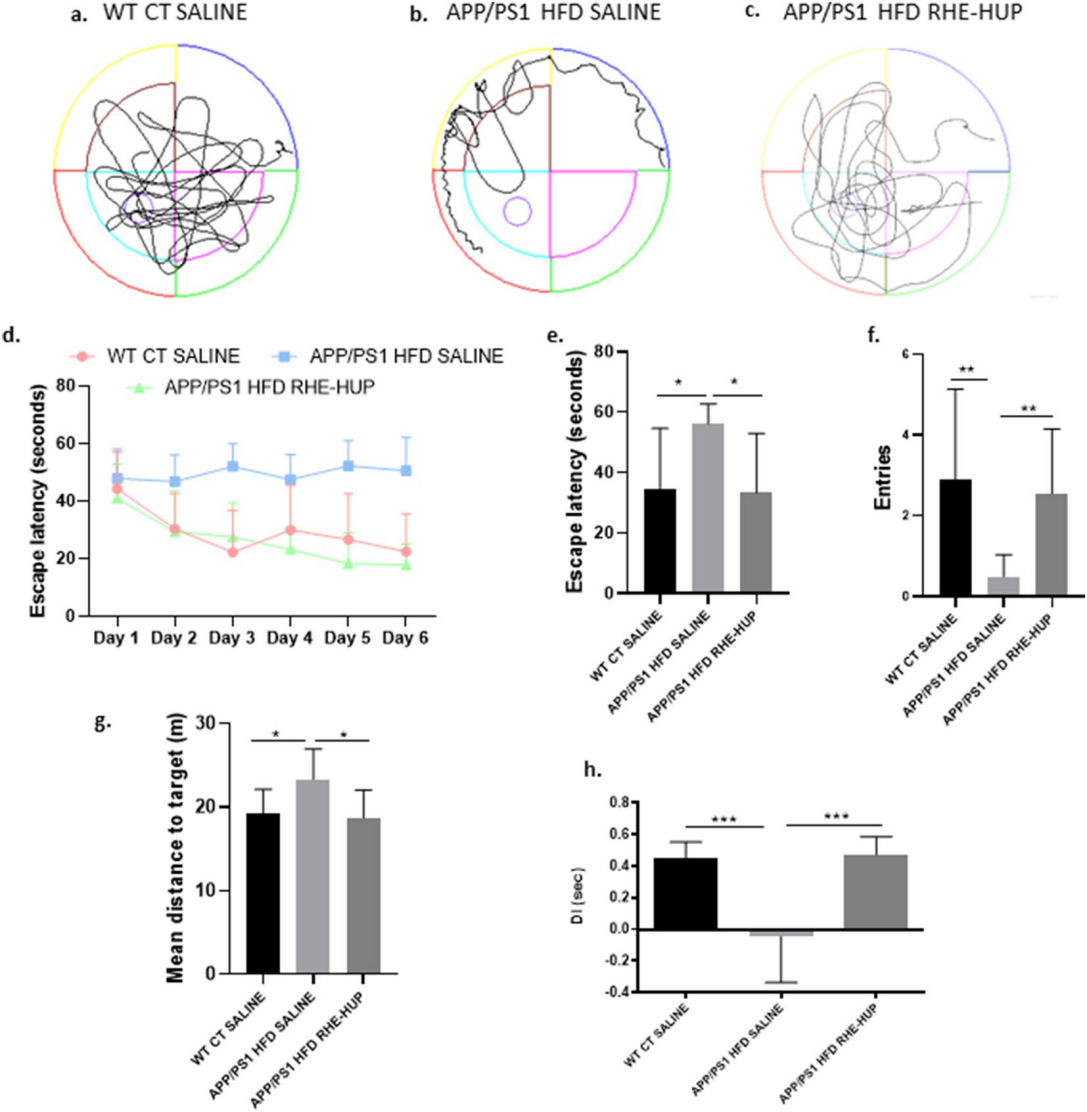
**a**–**c**. Representative swim paths on the memory test. Learning curves of MWM during the spatial acquisition phase **d** and escape latency **e**, entries in platform **f** and mean distance traveled **g** on test day (n = 13). One-way ANOVA and Tukey’s post- test were performed, except in the case of the analysis of entries in the platform where Krushal-Wallis was conducted. In all cases, * p < 0.05 and ** p < 0.01. **h**. NORT, Discrimination Index (DI) expressed in seconds (n = 13). Statistical analysis was performed by one-way ANOVA and Tukey post-test, *** p < 0.001

It has been described that one of the most important features of APP/PS1 mice is cognitive decline in terms of memory and spatial memory [54, 55]. To demonstrate the efficacy of RHE-HUP treatment in the recovery of cognitive decline, MWM and NORT tests were performed. Regarding MWM, APP/PS1 HFD SALINE mice showed an obviously more erratic trajectory, being unable to find the platform compared with WT CT SALINE mice. However, after RHE-HUP treatment, the trajectory of APP/PS1 HFD RHE-HUP tended to return to normality (Fig. 7a–c). In Fig. 7d, the escape latency of all groups throughout the training period is shown. The training performed by the different groups demonstrated an improvement of the learning ability in those animals treated with RHE-HUP in comparison to those treated with saline. In the same line, the results obtained on the test day showed a significant increase in escape latency in the APP/PS1 HFD SALINE when they were compared with control group (p < 0.05), effect which was reverted in those animals treated with the drug (p < 0.05) (Fig. 7e). Moreover, other parameters studied in the same test, such as the number of entries on the platform or the mean distance traveled to reach it, showed the same tendency toward improvement of cognitive function after RHE-HUP administration. Regarding the number of entries, the time of crossing through the target platform was significantly reduced in non-treated animals (p < 0.01), whereas after treatment that number was recovered, reaching similar values to WT CT (p < 0.01) (Fig. 7f). In the case of the mean distance traveled to find the platform, non-treated animals swam a longer distance compared to the control group (p < 0.05), while after treatment, they reached the platform more easily (p < 0.05) (Fig. 7g). In agreement, in the NORT APP/PS1 HFD SALINE mice presented a decreased DI compared to the control group (p < 0.001), whereas the DI was recovered after treatment (p < 0.001), clearly indicating that RHE-HUP rescued mice from the memory deficit observed in this pathological model (Fig. 7h).

## Discussion

AD is nowadays recognized as a multifactorial and heterogeneous disease in which metabolic alterations play an important role [56–58]. Previous work has shown that RHE-HUP improves the main hallmarks of AD in APP/PS1 mice [35]. However, the effect of RHE-HUP in an AD familial model of mice with a metabolic syndrome-like was not evaluated, yet. Our results demonstrated that RHE-HUP significantly reduces neuroinflammation, Aβ deposition and Tau phosphorylation, considered some of the main underlying disease mechanisms. Additionally, RHE-HUP treatment succeeded in increasing the levels of BDNF and other synapse-related proteins in the brain, which resulted in an increase in the number of dendritic spines, improving memory and learning. However, these changes were not associated with modifications in the metabolic peripheral parameters.

HFD consumption leads to metabolic alterations, including insulin resistance and T2DM [59, 60], both conditions frequently associated with the development of dementia [41, 61]. T2DM is a complex disorder that begins with a state of insulin resistance, leading to hyperinsulinemia and hyperglycemia, which is known to cause different alterations in the brain. Our study confirmed that HFD induces an increase in body weight, hyperglycemia and insulin resistance in APP/PS1 mice accompanied by the downregulation of IRS2 protein levels in the liver, a protein involved in insulin signaling regulation. However, the treatment with RHE-HUP did not reverse these effects, leading us to the conclusion that the observed benefits provided by RHE-HUP may not be due to a peripheral metabolic regulation, rather to a central effect. One of the possible answers could be that this molecule was designed to hit multiple targets involved in the pathogenesis of AD, i.e., to reach biological targets located at the central nervous system. Indeed, studies performed in *parallel artificial membrane permeability assays for blood-brain barrier* (PAMPA-BBB) clearly demonstrated that this compound was able to enter the brain [34]. This fact was supported by the results obtained in previous studies where a reduction of Aβ levels and Tau phosphorylation leading to a memory amelioration, was observed after chronic administration of RHE-HUP to APPswe/PS-1dE9 mice [35]. Moreover, ex vivo [62–64] and in vivo biodistribution [65] studies with other hybrid compounds, closely related to RHE-HUP in terms of chemical structure and physicochemical properties, have demonstrated that this type of compounds readily enters the brain, some of them with more favorable brain/plasma ratio than the most prescribed anti-Alzheimer drug donepezil [65]. Very likely, this could be also the case for RHE-HUP, which might account for its preferential central vs peripheral effects observed in this work using the familial AD mouse model, challenged by high-fat diet.

Brain insulin, apart from controlling energy metabolism, is also involved in other multiple functions including synaptogenesis, synaptic remodeling, and neurotransmitter level modulation. Thus, unbalanced insulin signaling, and metabolism may lead to cognitive decline and AD [66]. IRS2, a major component of the insulin/insulin-like growth factor-1 signaling pathway and a key factor in T2DM, also has a role in synaptic plasticity, learning and memory. A study carried out by Tanokashira and colleagues found that young adult C57BL/6 J mice lacking IRS2 displayed hippocampus-associated behavioral alterations due to IRS2 deficiency-induced impairments of brain energy metabolism [67]. Our results agree with these data, since a IRS2 reduction was observed in the APP/PS1 HFD SALINE group recovering its levels after the RHE-HUP treatment. It has been also described that IRS2 signaling promotes the dephosphorylation of Tau, suggesting that failure on this pathway could lead to an hyperphosphorylation of Tau protein, considered one of the main early mechanisms of AD. Therefore, Tau phosphorylation might be a direct consequence of reduced insulin–IGF signaling during aging [47, 68]. Likewise, one of the main kinases responsible for Tau phosphorylation is GSK3β [69]. The phosphorylation of this kinase in Tyr216 leads to its own activation which results not only in the increase in Tau phosphorylation levels [70], but also contributes to neuronal death independently of Tau [71]. In agreement with this, the present study demonstrated that RHE-HUP administration significantly reduced Tau phosphorylation, by IRS2 and p-GSK3β regulation, which could explain the restoration of dendritic spine number and the resulting behavioral improvement observed in A PP/PS1 HFD mice after the treatment.

In addition to hyperphosphorylated Tau, another well-known hallmark of AD is the accumulation of β-amyloid deposits. Several studies have interconnected both processes defining Aβ plaques as the main triggers of Tau hyperphosphorylation and Tau tangle formation, as a result of an imbalance between Aβ production and Aβ clearance [14, 72]. In agreement with these previous data, we observed a significant reduction in the number of hippocampal and cortical Aβ plaques induced by RHE-HUP due to BACE1 inhibition. In turn, this correlated with the reduction of the levels of Aβ_42_, the most hydrophobic and aggregation-prone form of this peptide and, the predominant one in senile plaques [73, 74]. This event also explained the reduction in hyperphosphorylated Tau observed in this group.

As described by Pérez-Areales and coworkers, RHE-HUP seems to inhibit AChE [38], a prime target in AD, since the cholinergic deficit has been widely observed in AD patients and is directly responsible for the cognitive decline [75, 76]. However, the importance of this enzyme in the disease goes much further, since it has been described that it might bind to Aβ and promote its deposition [77], turning the combination of AChE + Aβ into more toxic to cells than Aβ alone [78].

Taking all this into account and according to our findings, the effect of RHE-HUP on decreasing the Aβ production and subsequent accumulation might be attributed to four main factors: (i) the inhibition of AChE, avoiding the interaction with Aβ and the consequent formation of the toxic aggregates; (ii) the inhibition of the amyloidogenic pathway by decreasing hippocampal BACE1 activity; (iii) the direct reduction of APP protein levels and (iv) the activation of the non-amyloidogenic pathway by increasing ADAM10 levels [79–83]. In addition, our results show that RHE-HUP treatment increased IDE levels in the hippocampus, an enzyme that not only participates in Aβ elimination, but also plays a key role in insulin degradation, all together contributing to a reduction in Aβ deposition and cognitive improvement [84].

The glial activation in the brain is also an important pathological feature of neurodegenerative diseases, including AD [85–87]. Although early in the disease neuroinflammation may represent a protective response, an excessive reaction can cause or contribute to the pathology [88]. Several reports have described that the presence of Aβ and Tau hyperphosphorylation activate microglia and astrocytes [89–91], demonstrating that microglia can play dual roles in Aβ pathogenesis. Microglia may help to eliminate Aβ aggregation, and it may facilitate Aβ accumulation through the release of neurotoxic proteases and pro-inflammatory factors, which contribute to the neuroinflammation [92–96]. Thus, it generates a vicious circle in which Aβ plaques potentiate the release of inflammatory molecules and, at the same time, these molecules stimulate the formation and accumulation of Aβ [97, 98]. Moreover, it is well-known that the chronic consumption of HFD increases stress in different pathways including neuroinflammation [99], contributing to the development of cognitive impairment. In this line, Wieckowska-Gacek et al. demonstrated that 4-months-old APPswe transgenic mice fed with western diet exhibited such brain neuroinflammation and accelerated amyloid pathology comparable to that induced by the administration of pro-inflammatory lipopolysaccharide (LPS). Hence, it highlighted the role that diet can play in neuroinflammation and, consequently, in AD [100]. In this sense, the observed decrease in the activation of microglia and astrocytes after RHE-HUP treatment might be due to the reduction in Tau phosphorylation and in Aβ deposition, but also to the improvement in the insulin signaling pathway at the central level observed upon treatment. Toll-like receptors play a pivotal role in brain injury and neurodegeneration, and, in CNS, they are mainly expressed in glial cells [101]. Specifically, the activation of TLR4 triggers the downstream stimulation of the nuclear factor kappa-light-chain-enhancer of activated B cells (NFK-β) and the induction of genes that encode inflammation-associated molecules and cytokines, such as IL-6 and TNF-α [102, 103]. Furthermore, it has been demonstrated that TLR4 deficiency protects against ethanol-induced glial activation, induction of inflammatory mediators, and apoptosis [101]. For this reason, the attenuation of the neuroinflammation observed after the RHE-HUP treatment could be related with the decrease of TLR4 levels, in agreement with previous studies which demonstrated that the treatment with resveratrol attenuated the increase in protein levels and the downstream activation of the pathway [104, 105].

In the same way, PTP1B also demonstrated a significant decrease in the RHE-HUP treated mice. Several studies have reported that the inhibition of PTP1B favors the inactivation of unfolded protein response (UPR) and neuroinflammation, thereby protecting against cognitive decline [106]. For this reason, PTP inhibitors have been suggested as a promising therapeutic modulation of microglial activation in neuroinflammatory diseases, including AD [107]. In addition, PTP1B not only has been related to this group of pathologies, but also represents a convergent point between AD and T2DM. In fact, preclinical studies have demonstrated that mice lacking PTP1B were resistant to weight gain and remained sensitive to insulin after HFD consumption [108, 109] suggesting that PTP1B downregulation could be key in order to improve the features observed in AD pathogenesis by the regulation of insulin signaling pathway and neuroinflammatory processes [110].

Moreover, in a pathological environment the released cytokines and chemokines contribute to an excessive pruning of synaptic terminals causing synaptic dysfunction and neuronal loss [111]. In fact, another important pathway in which PTP1B is involved is the BDNF/TrkB pathway [112]: PTP1B down-regulates neuronal BDNF-TrkB pathway, whereas the PTP1B inhibition stimulates BDNF signaling [113, 114]. Considering that preclinical studies suggest that the increase in BDNF levels is a suitable strategy to enhance the cognitive process [115], the decrease in PTP1B levels induced by RHE-HUP treatment observed in our results and the consequent increase in BDNF levels could explain the recovery in dendritic spines number caused by the treatment. In addition, dendritic spines loss is also related with Aβ and Tau pathology, since a study performed by Bittner et al., demonstrated that mice coexpressing mutant APP, PS1 and Tau, presented a strong loss of dendritic spines with accumulation of hyperphosphorylated Tau protein as well as soluble Aβ [116]. Therefore, the reduction already discussed in Aβ accumulation and Tau hyperphosphorylation caused by the treatment might also be contributing to the recovery of dendritic spines. These results were also accompanied by an increase in DBN1 levels. DBN1 is typically located in postsynaptic regions of excitatory synapses, and it is responsible for controlling spine function and morphology [117, 118]. Its preservation has been related to neuroprotection, and, by contrast, its reduction in the hippocampus has been linked to cognitive deficits [119, 120]. Thus, our data confirm that the increase in DBN1 could be associated with the improvement observed in cognitive functioning. In the same way, synaptophysin and neurexin showed a similar profile. Synaptophysin is a glycoprotein present in synaptic vesicles which is related to synaptic plasticity. Thus, a decrease in its levels has been related to cognitive impairment [121]. At the same time, neurexin downregulation has also been associated with cognitive impairments since it has been found to be active in synapse maturation and adaptation of synaptic strength [122]. In addition, it has been demonstrated that Aβ_42_ oligomers bind to neurexin, and this interaction leads to a decrease in its expression, inducing synapse pathology [123]. This would explain the increase in neurexin protein levels produced by the decrease in Aβ_42_ levels observed after treatment with RHE-HUP.

Recent postmortem studies in people with AD have shown that the number of dendritic spines is lower in patients with clinically evident AD compared to controls, and similar between control subjects and subjects that are cognitively normal but present the underlying biological features of AD. Thus, these observations provide cellular evidence supporting the hypothesis that dendritic spine plasticity provides a mechanism of cognitive resilience that protects people with an early stage of dementia from developing AD [124, 125]. In fact, numerous preclinical studies have related the loss of dendritic spines with hippocampus-dependent learning and memory ability impairments [126–128]. In the present study, RHE-HUP treatment induced the recovery in the number of dendritic spines, which was accompanied by an improvement in hippocampal-dependent recognition memory assessed by NORT, as well as spatial and learning memory evaluated by MWM.

**Fig. 8:**
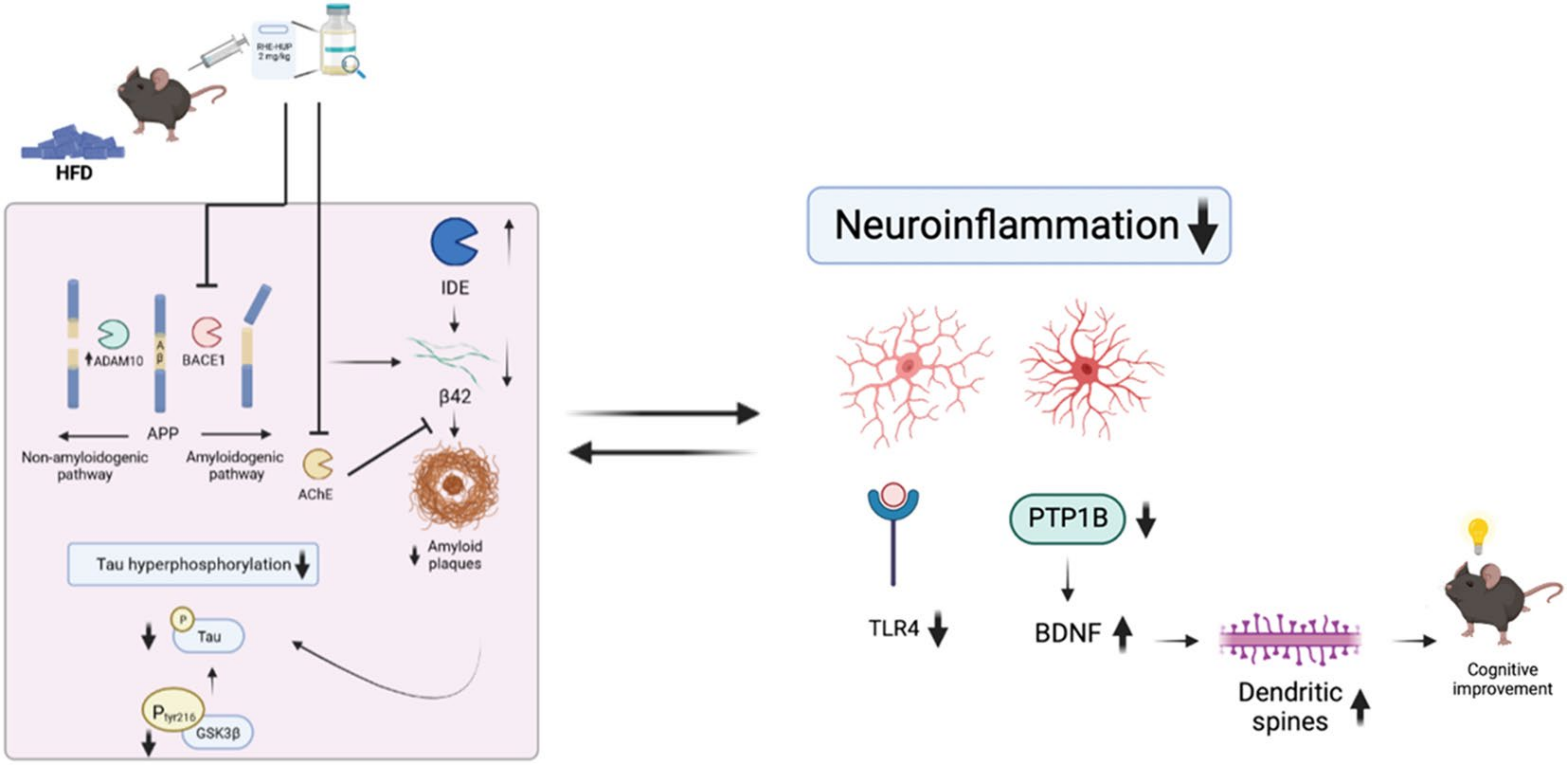
Schematic representation of the effects of RHE-HUP treatment in APP/PS1 mice fed with HFD. The figure shows the pathological mechanisms targeted by RHE-HUP that could explain the improvement in cognition observed in this double pathological model

In conclusion, the present study demonstrates that the multi-target compound RHE-HUP restores the number of dendritic spines and enhances cognition in APP/PS1 mice, whose pathology is exacerbated with HFD consumption, by regulation of brain insulin signaling and neuroinflammation, which contributes to the reduction of hyperphosphorylated Tau and Aβ levels (Fig. 8). However, we did not observe peripheral metabolic regulation induced by the drug administration, suggesting that the improvement observed in our model is exclusively due to a regulation at central level. These results support RHE-HUP as a new promising molecule for the treatment of AD, also in those individuals with metabolic disturbances.

## Data Availability

All data generated or analyzed during this study are included in this published article.

## Abbreviations

Aa: Amino acids
Aβ: Amyloid-beta
AChE: Acetylcholinesterase
AD: Alzheimer’s disease
APP: Amyloid precursor protein
APP-CTF: APP-C-terminal fragment
BACE1: β-Secretase
BDNF: Brain derived neurotrophic factor
DBN1: Drebrin 1
DG: Dentate gyrus
DI: Discrimination index
GFAP: Glial fibrillary acidic protein
(p)-GSK3β: (Phospho)-Glycogen synthase kinase-3β
GTT: Glucose tolerance test
HFD: High-fat diet
IBA1: Ionized calcium-binding adapter molecule 1
IDE: Insulin-degrading enzyme
IRS2: Insulin receptor substrate protein 2
ITT: Insulin tolerance test
LPS: Lipopolysaccharide
LTP: Long-term potentiation
MWM: Morris water maze
NFKβ: Nuclear factor kappa-light-chain-enhancer of activated B cells
NFTs: Neurofibrillary tangles
NORT: Novel object recognition test
PB: Phosphate buffer
PBS: Phosphate-buffered saline
PTP1B: Protein tyrosine phosphatase
RHE-HUP: Rhein-huprine
sAPPβ: Soluble amyloid precursor protein β fragment
SD: Standard deviation
TLR4: Toll-like receptor 4
T2DM: Type 2 diabetes mellitus
UPR: Unfolded protein response
WT: Wild-type
